# Graft and Button Modification of Technique of Coracoclavicular Joint Reconstruction in Treatment of Chronic Type V Acromioclavicular Joint Dislocation: A Case Report

**DOI:** 10.5704/MOJ.1907.009

**Published:** 2019-07

**Authors:** YC Leong, J Muhammad-Suhairi

**Affiliations:** Department of Orthopaedics, Hospital Sultanah Bahiyah, Alor Setar, Malaysia

**Keywords:** AC joint reconstruction, gracilis graft, running whip stitch

## Abstract

Treatment of chronic Rockwood’s type V Acromioclavicular (AC) joint dislocation remains controversial. We describe a surgical technique to reduce and maintain AC joint using a combination of gracilis autograft with GraftMax™ button (Conmed Inc, Utica, NY). Graft was prepared using running whip stitch technique with No. 5 Hi-Fi high strength suture (ConMed Linvatec, Largo, FL). Our technique reduces intraoperative clavicular and coracoid tunnel fracture and restores anatomical coracoclavicular ligament. At sixth week and third month postoperatively, the patient demonstrated good clinical and radiographic outcome.

## Introduction

To date there is no consensus on gold standard treatment for chronic Rockwood’s type V acromioclavicular (AC) joint dislocation. We report an open surgical technique, early clinical and radiographical outcomes of AC joint reconstruction using autologous gracilis tendon grafts and Graftmax^TM^ button.

## Case Report

**Fig. 1:**
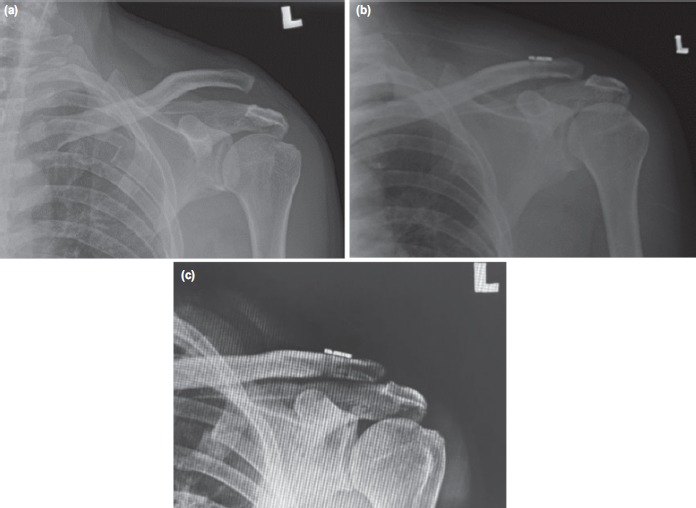
(a) Preoperative anteroposterior radiograph of left shoulder showing Rockwood’s grade V AC joint dislocation. (b) Immediate postoperative radiograph showing left AC joint reduced. (c) At 6 week follow-up, left AC joint reduction was maintained, no graft or hardware failure noted.

A 63-year old motorcyclist presented to our center one day after a motor vehicle accident. He complained of left shoulder pain and inability to abduct his left shoulder. His left shoulder contour was asymmetrical with left acromioclavicular joint tenderness. No neurological deficit was noted. Plain radiograph revealed left acromioclavicular joint dislocation, Rockwood’s type V (Fig. 1a).

Following the diagnosis, left acromioclavicular joint reconstruction using autologous gracilis graft was planned to reduce the acromioclavicular joint and improve his shoulder function.

**Fig. 2:**
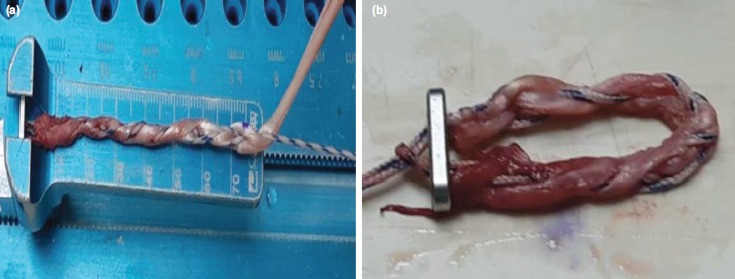
(a) Gracilis autograft was prepared using No. 5 Hi-Fi high strength (ConMed Linvatec, Largo, FL). The graft was prepared using whip stitch technique. (b) The prepared graft was later secured with GraftMaxTM button (Conmed Inc, Utica, NY).

At week five, on the next available operating date, the procedure was performed under general anaesthesia. Ipsilateral gracilis tendon was harvested and prepared by running whip stitch technique using No.5 Hi-Fi high strength sutures (ConMed Linvatec, Largo, FL) (Fig. 2a). Left acromioclavicular joint and distal clavicle were exposed subperiosteally up to the level of the coracoclavicular ligament. The coracoid process and its conjoint tendon were exposed inferiorly by dissecting the deltopectoral fascia. Conjoint tendon was retracted medially and lateral aspect of coracoid process was prepared for graft passage.

**Fig. 3:**
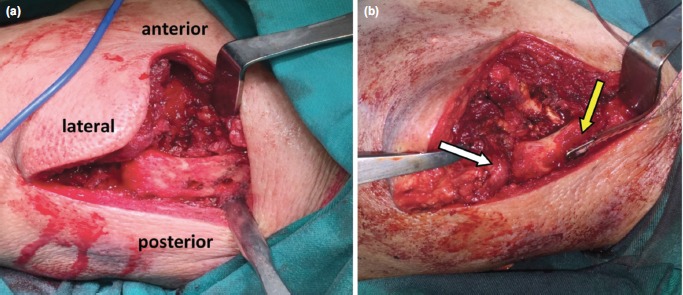
(a) Two clavicular tunnels, around 1 cm apart drilled around 2.5cm lateral to the acromioclavicular joint to restore anatomical position of conoid and trapezoid ligament. (b) The graft was secured with GraftMaxTM button (Conmed Inc, Utica, NY) (yellow arrow). Intraoperative assessment of reduced acromioclavicular joint showed no anteroposterior and superoinferior translation (white arrow).

The base of the coracoid process was drilled using Schanz pin size 4mm in superior to inferior direction, followed by another two clavicular tunnels, around 1 cm apart, drilled 2.5cm lateral to the acromioclavicular joint (Fig. 3a). The graft length was estimated by looping a suture through the coracoid and both clavicular tunnels. The gracilis graft was passed through the tunnel using a suture-passing device. Intraoperative reduction was confirmed clinically and radiologically. Both ends of the graft were also secured with the GraftMax™ button (Conmed Inc, Utica, NY) (Fig. 3b). The deltopectoral fascia was repaired with absorbable suture. Postoperatively, patient was put on arm sling for six weeks. Pendulum exercise was initiated three weeks after the procedure. Arm sling was removed at six weeks. The patient continued the prescribed rehabilitation program to achieve full range of motion of the shoulder.

Satisfactory clinical and radiological outcome were achieved at six weeks and three months postoperative (Fig. 1c). Functional outcome was assessed using Oxford shoulder score and constant shoulder score. The resultant Oxford shoulder score was 43 at sixth week and third month postoperation, and the constant shoulder scores were 81 and 87.

## Discussion

High grade acromioclavicular joint dislocation (Grade IV and above) requires surgical intervention to prevent painful shoulder joint movement. The available surgical options include acromioclavicular joint fixation (hook plates), coracoclavicular fixation (Bosworth screw, mersilene tape, tightrope), and ligament reconstruction (Weaver and Dunn, anatomical coracoclavicular joint reconstruction)^1^.

Bosworth screw and hook plate fixation provide rigid AC joint fixation but are often related to hardware failure requiring hardware removal^1^. Meanwhile, tightrope system (Arthrex, Naples, FL) and mersilene tape (Ethicon, Somerville, NJ) serve as a non-rigid fixation that secure superoinferior translation but not anteroposterior translation. AC joint reconstruction was historically used to treat chronic AC joint instability. The earlier technique such as Weaver and Dunn procedure exploits coracoacromial ligament for AC joint reconstructions but demonstrated only 25% of biomechanical strength as compared to intact coracoclavicular ligament, which resulted in up to 30% of failure rate^2^.

Anatomical coracoclavicular joint reconstruction using GraftRope (Arthrex Inc. Naples, FL) has shown equivalent biomechanical strength as the native intact ligament^3^. However, Cook *et al* reported up to 80 % loss of reduction in patients treated with GraftRope at the mean of seven weeks^4^. In addition, several studies have also shown significant increased risk of clavicular fracture in clavicular tunnels of 5mm or greater in diameter^5^. Interference screw used to secure graft end may increase risk of tunnel fracture. Our population has smaller clavicle size and is at greater risk of clavicular fracture. Therefore, we developed a modified reconstruction technique to prevent clavicular tunnel fracture. Both clavicular and coracoid tunnel size 4mm were prepared as compared to previous 5mm size. We introduced the concept of hybrid graft by preparing the graft using running whip stitch technique as compared to previous technique that only sutured both tail end of graft. Looping our graft through coracoid tunnels will reduce the motion of graft bone interface and thus tunnel widening as reported in previous case series^4^.

The limitations of our surgical technique are graft length estimation, large surgical wound, and short-term follow-up. The graft is elastic and tends to crumple during passage through the tunnel. We successfully passed the graft through only after multiple attempts. The large surgical wound in our case gave us better access to the anatomy (Fig. 3). With better understanding of anatomy and the use of image intensifier, this procedure can be performed by mini-open or arthroscopy assisted. Long- term follow-up is also needed to look at the clinical and radiological outcome.

In conclusion, positive outcome is achieved using the modified technique of AC joint reconstruction in treating Rockwood’s type V chronic AC joint dislocation. Patient outcome may also improve if the procedure is conducted using mini open or arthroscopy assisted technique.
